# Evaluation of knowledge levels amongst village AIDS committees after undergoing HIV educational sessions: results from a pilot study in rural Tanzania

**DOI:** 10.1186/1472-698X-11-14

**Published:** 2011-12-13

**Authors:** Elizabeth J Epsley, Benjamin Nhandi, Alison Wringe, Mark Urassa, Jim Todd

**Affiliations:** 1 Faculty of Epidemiology and Population Health, London School of Hygiene and Tropical Medicine, London, UK; 2 TAZAMA Project, National Institute of Medical Research, Mwanza, Tanzania

## Abstract

**Background:**

Village AIDS committees (VAC) were formed by the Tanzanian government in 2003 to provide HIV education to their communities. However, their potential has not been realised due to their limited knowledge and misconceptions surrounding HIV, which could be addressed through training of VAC members. In an attempt to increase HIV knowledge levels and address common misconceptions amongst the VACs, an HIV curriculum was delivered to members in rural north western Tanzania.

**Methods:**

An evaluation of HIV knowledge was conducted prior to and post-delivery of HIV training sessions, within members of three VACs in Kisesa ward. Quantitative surveys were used with several open-ended questions to identify local misconceptions and evaluate HIV knowledge levels. Short educational training sessions covering HIV transmission, prevention and treatment were conducted, with each VAC using quizzes, role-plays and participatory learning and action tools. Post-training surveys occurred up to seven days after the final training session.

**Results:**

Before the training, "good" HIV knowledge was higher amongst men than women (p = 0.041), and among those with previous HIV education (p = 0.002). The trade-centre had a faster turn-over of VAC members, and proximity to the trade-centre was associated with a shorter time on the committee.

Training improved HIV knowledge levels with more members achieving a "good" score in the post-training survey compared with the baseline survey (p = < 0.001). The training programme was popular, with 100% of participants requesting further HIV training in the future and 51.7% requesting training at three-monthly intervals.

**Conclusions:**

In this setting, a series of HIV training sessions for VACs demonstrated encouraging results, with increased HIV knowledge levels following short educational sessions. Further work is required to assess the success of VAC members in disseminating this HIV education to their communities, as well as up-scaling this pilot study to other regions in Tanzania with different misconceptions.

## Background

Misconceptions, myths and stigmas surrounding HIV/AIDS are rife in many sub-Saharan African settings, hindering HIV education [1-6]. Several studies in rural Tanzania have identified an array of constantly evolving misconceptions associated with HIV transmission, condoms and antiretroviral therapy (ART) [7,8]. HIV education is further complicated by the pervasive stigma that surrounds infection in many African settings [9].

Witchcraft is frequently attributed to be the cause of HIV/AIDS, with many believing that it can be cured by traditional medicine alone, or accompanied by spiritual healing [10]. The reasons for this are complex, and partially reflect the pervasive stigma surrounding HIV infection, which creates an environment that is conducive to transferring blame away from an individual's own behaviour, towards an external power, so that the infected individual becomes a victim of a malevolent third party, rather than being responsible for his/her own actions [11]. Furthermore preferences for traditional healers over health facilities are common due to familiarity, trust, accessibility and their cheaper cost [12].

Another common misconception in some rural areas of Tanzania is that the lubricant oil present on condoms contains the HIV virus, such that their use propagates, rather than prevents, the spread of the epidemic [13]. Furthermore, these rumours are sometimes accompanied by claims that the HIV virus has been deliberately placed there by white people to infect and kill Africans; or that they knowingly distribute old and inferior products [13]. In addition it has been reported by some individuals that both the man and the woman should use a condom during sexual intercourse, suggesting that even when they are considered acceptable, their effectiveness may be compromised by a lack of correct knowledge about their use [13].

The existence of myths and misconceptions around HIV occurs in the context of treatment, as well as in relation to the aetiology of infection and prevention interventions. ART is provided free of charge in Tanzania and has been available in Mwanza city since 2005 and Kisesa Health Centre since 2008 [14]. Following the introduction of free ART, some HIV-related stigmas have declined, but others have emerged in their place, including the perception among some community leaders that improvements in ART patients' health has led to increased sexual activity, further spreading HIV [3]. Another misconception recorded in a separate study was that engaging in sex whilst on ART accelerated death, and that the use of the drugs requires sexual abstinence [10]. The mistrust and confusion associated with ART may discourage the initiation of treatment for infected individuals and may lead to de-motivation among those already enrolled in care and treatment programmes [10]. Furthermore, since decision-making with regards to ART commonly reflects the views of sexual partners, family members and friends as well as influential community members, misconceptions around ART within these social networks can either generate support or discouragement for sustained adherence to therapy [10].

One potential vehicle for providing communities with HIV education, and for addressing the common misconceptions that can undermine prevention and treatment programmes are village AIDS committees (VACs). VACs were formally established in 2003 in Tanzania, in order to give local governments responsibility for reducing HIV prevalence [15,16]. The aim of the VACs is for its members to disseminate information to the community on various aspects of HIV transmission, prevention and treatment, in order to increase understanding about HIV amongst the local community [15]. Each village in Tanzania is required to have its own committee consisting of approximately 15 members. Recruitment of VAC members is based upon Tanzanian policy, with national guidelines stating that the VAC should include both men and women, and should consist of a diverse range of individuals including prominent citizens from the village council, youth representatives, religious representatives and residents living with HIV/AIDS [15]. Any monitoring and evaluation work that may have been conducted previously with VACs in Tanzania is undocumented.

However, VACs are unlikely to be able to meet their responsibilities unless they are equipped with comprehensive HIV education and an explanation of their expected roles. HIV curricula have previously been piloted in a variety of settings, often targeting young people prior to sexual debut. Mema Kwa Vijana (MKV) was a curriculum implemented and evaluated in 62 primary schools in rural Mwanza between 1999 and 2001 [17]. This teacher-led and peer-assisted programme incorporated various methods including scripted dramas, role-plays and stories to educate students on aspects of sexual health. School-based HIV curricula have since been expanded to other areas within Tanzania and elsewhere in sub-Saharan Africa [18-20]

Despite the success of school-based interventions, they are unlikely to reach many sexually active members of the community with poor levels of knowledge about HIV and at risk of HIV infection. Interventions that are delivered in a community setting have the potential to target higher risk youths who have dropped out of school, as well as reaching the rest of the village population [21]. The development of curricula focusing on sexual health, HIV and STIs targeting people in schools, clinics or in community settings have been shown to be promising interventions towards reducing risky behaviours [22-24]

This paper describes the evaluation of a tailored HIV curriculum for VACs within a rural area in north-west Tanzania, with the aim that this knowledge could be disseminated to the wider community. A key objective of this pilot study was to evaluate whether the curriculum increased levels of HIV knowledge among the VAC members by conducting baseline and post-training surveys. A further objective was to ascertain whether the demographic composition of the VAC across the villages conformed to Tanzanian guidelines.

## Methods

### Study setting

Kisesa ward, located 20 km from Mwanza, Tanzania, consists of a trading centre and six villages, defined as either remote-rural or roadside [25]. A large cohort study was established in 1994 with demographic surveillance taking place on a six monthly basis and serological surveillance occurring every three years, in order to monitor the dynamics of the HIV epidemic and more recently to evaluate the impact of government-provided HIV prevention and treatment activities. In 2007 in round 5 of the serological surveillance the population of the ward was approximately 30,000. In 2007, HIV prevalence within Kisesa ward was 6% among men and women, with disparities between roadside and remote villages as well as between age-groups.

Kisesa ward has experienced several government-led HIV interventions in the area including a school-based HIV education programme, condom promotion and a voluntary counselling and testing (VCT) service [25]. VACs have been operating in some capacity within Kisesa ward for the past sixteen years. However until 2009, VAC members had not been provided with any formal HIV training as part of their participation on the committee.

### Design of the baseline and post-training surveys

The key aim of the baseline and post-training surveys was to evaluate any improvement in HIV knowledge levels amongst VAC members after HIV training had been delivered. The baseline survey captured information on the socio-demographic characteristics, HIV knowledge levels of VAC members, and their perceptions on the development of the proposed curriculum.

Knowledge levels were assessed through true or false questions addressing misconceptions that had been previously identified in the study area, as well as questions focusing on HIV transmission and access to ART, and are shown in Table 1. Several questions were adapted from the extensive serosurvey questionnaire to assess the uptake of VCT, knowledge of HIV transmission and presence of local misconceptions regarding HIV prevention and treatment [25]. Only one question in the baseline questionnaire, on the knowledge of the availability of free ART in the health centre, was identical to the serosurvey question allowing direct comparison between the VAC members and the general population.

**Table 1:** Questions used in the baseline and post-training questionnaires, and the overlap with the 2007 serosurvey questionnaire

	Question found in:		
	**Baseline questionnaire**	**Post-training questionnaire**	**Serosurvey questionnaire**

**Do you know what causes HIV/AIDS?** **Mention all the ways you know:** **Having sex with casual/high risk partner** **Having sex without a condom** **Unsafe blood transfusion** **Unsterile injections** **Mother to child transmission** **Incisions on the body** **(Mosquitoes)** **(Witchcraft)**	Yes	Yes	Adapted

**Can AIDS be transmitted by sharing cups and plates?**		Yes	Identical

**Can AIDS be transmitted by kissing?**		Yes	Identical

**Is it possible for a healthy looking person to have HIV/AIDS?**		Yes	Identical

**Would you recommend a friend to have VCT?**	Yes	Yes	Identical

**ART prolongs life if taken consistently**	Yes	Yes	Adapted

**ART drugs are available free of charge in Tanzania**	Yes	Yes	Identical

**Inadequate nutrition whilst on ART accelerates death**	Yes	Yes	Adapted

**Condoms have the virus on them**	Yes	Yes	Adapted

**HIV is only found in the trade centre**	Yes	Yes	

**HIV symptoms can be cured by traditional/spiritual healing**	Yes	Yes	

**It is necessary to use condoms when engaging in sexual activity when on ART**		Yes	

**HIV can be transmitted from mother to child during pregnancy/birth or breastfeeding**		Yes	

**Circumcision alone prevents HIV infection**		Yes	

**It is possible to become infected with HIV even if you have only had sex once**		Yes	

Baseline surveys were conducted in four of the six villages in Kisesa ward, however only three of the villages were involved in training and post-training surveys due to time constraints. One of the roadside villages (Isangijo) was excluded at this stage. All members of the VAC were invited to attend and invitations were delivered two days prior to the survey. Post-training surveys occurred one week after the HIV training in three villages.

### The HIV curriculum

The HIV curriculum was designed using previous training programmes implemented in sub-Saharan Africa, aided by MKV materials provided by the AMREF office in Mwanza and other online resources [17] These resources provided the basis for the VAC curriculum. Sections of the MKV programme were modified and tailored, by the first two authors, in accordance with Kisesa ward's misconceptions which were identified in the baseline survey, and from previous studies in the local area [3,7,8,10-13]

The VAC curriculum was designed to focus on 3 areas; [i] HIV transmission, [ii] HIV prevention and [iii] ART (3,10,11). Three villages were selected for training including a remote-rural and a roadside village, along with the trade centre. All members of the VAC were invited to attend and were compensated TSH 5000 (approximately 4 USD) for loss of income and travel costs incurred. Contact information was collected from attendees at the training programme.

The three topics were delivered over three two-hour sessions in KiSwahili by a Tanzanian research assistant, with role-plays to address misconceptions associated with condoms and ART and quizzes to consolidate knowledge at the end of each session. Small group activities encouraged VAC members to ask questions and raise issues, and ensured maximum participation. A training manual supplemented the programme and was left with VAC members after completion of the training as reference material to guide their own sessions with the community.

### Quantitative methods

Survey data were entered in Epi-data and analysed using Stata 10 (StataCorp, College Station, Texas, USA). An overall HIV knowledge indicator was created by adding the number of correct answers to the "true or false" questions. A cut off for indicating good knowledge (against poor knowledge) was taken at the median number of correct responses, which was 10 in the baseline survey (range 6-14). Cross tabulations and chi square tests were used to assess associations between the variables. Logistic regression was used to obtain odds ratios (OR) and 95% confidence intervals (95%CI) for factors associated with HIV knowledge levels. Serosurvey data from 2007 was used in the analysis to compare each village with the respective VAC knowledge on the availability of free ART treatment in Tanzania.

### Ethical approval

Ethical approval for the research was approved by the London School of Hygiene and Tropical Medicine and the Tanzanian Medical Research Coordinating Committee.

## Results

Participation rates were lower in the trading centre compared to the rural and roadside villages at every stage of the study: baseline survey, HIV training and post-training survey (Table 2).

**Table 2:** Participation rates in the study by sex and village

Village	Roadside Isangijo		Rural Welamasonga		Roadside Igekemaja		Trade Centre Kisesa		Total	
	**n/15**	**%**	**n/15**	**%**	**n/15**	**%**	**n/15**	**%**	**n/60**	**%**

**Baseline survey attendance**	6	40.0	12	80.0	8	53.3	5	33.3	31	51.7

**Males**	5	83.3	6	50.0	5	62.5	3	60.0	19	31.7

**Females**	1	16.7	6	50.0	3	37.5	2	40.0	12	20

**Training attendance**	*	*	14	93.3	15	100.0	10	66.6	39	86.7

**Males**	*	*	9	64.3	11	73.3	7	70.0	27	60

**Females**	*	*	5	35.7	4	26.6	3	30.0	12	26.7

**Post-survey attendance**	*	*	13	86.6	11	73.3	5	33.3	29	64.4

**Males**	*	*	9	69.2	7	63.6	5	60.0	21	46.7

**Females**	*	*	4	30.8	4	36.4	2	40.0	10	22.2

* VAC members from the roadside village of Isangijo only participated in the baseline survey due to time constraints

### Baseline characteristics of participants

The characteristics of the 31 participants in the baseline survey are shown in Table 3. The mean age was 40.3 years (range 22-56 years), with only 12 (39%) females. 68% were Roman Catholic. The mean duration that members had lived in their village was 23.4 years, with a range of 2 to 48 years. Many VAC members had been present on the committee since its establishment, with the mean duration on the VAC being 6 years, and a longer duration observed in the most remote village.

**Table 3:** Demographics and HIV knowledge levels amongst VAC members, prior to the HIV training sessions

Village Name	Roadside Isangijo		Rural Welamasonga		Roadside Igekemaja		Trade Centre Kisesa		Total	
	**n/N**	**%**	**n/N**	**%**	**n/N**	**%**	**n/N**	**%**	**n/N**	**%**

Demographic										

**Sex**										

**Male**	5/6	83.3	6/12	50.0	5/8	62.4	3/5	60.0	19/31	61.3

**Female**	1/6	16.7	6/12	50.0	3/8	37.5	2/5	40.0	12/31	38.7

**Age**										

**Under 40**	1/6	16.7	10/12	83.3	4/8	50.0	4/5	80.0	19/31	61.3

**40+**	5/6	83.3	2/12	16.7	4/8	50.0	1/5	20.0	12/31	38.7

**Lived in village (years)**										

**1-12**	0/6	0	2/12	16.7	1/7	14.3	3/4	75.0	6/29	20.7

**13-30**	2/6	33.3	7/12	58.3	5/7	71.4	1/4	25.0	15/29	51.7

**31+**	4/6	66.7	3/12	25.0	1/7	14.3	0/4	0	8/29	27.6

**Duration VAC (years)**										

**1-6**	6/6	100	3/12	25.0	5/8	62.5	4/5	80.0	18/31	58.1

**7-16**	0/6	0	9/12	75.0	3/8	37.5	1/5	20.0	13/31	41.9

**Religion**										

**Roman Catholic**	-	-	10/12	83.3	3/8	37.5	4/5	80.0	17/25	68.0

**African Inland Church**	-	-	1/12	8.3	4/8	50.0	0	0	5/25	20.0

**Baptist**	-	-	0/12	0	1/8	12.5	1/5	20.0	2/25	8.0

**Other**	-	-	1/12	8.3	0/8	0	0	0	1/25	4.0

**Previous HIV education**										

**No**	4/6	66.7	0/12	0	2/8	25	0/5	0	6/31	19.4

**Yes**	2/6	33.3	12/12	100	6/8	75	5/5	100	25/31	80.6

**Previous HIV training**										

**No**	3/6	50	3/12	25	1/8	12.5	2/5	40	9/31	29.0

**Yes**	3/6	50	9/12	75	7/8	87.5	3/5	60	22/31	71.0

**Ever had VCT**										

**No**	2/6	33.3	2/12	16.7	2/8	25	4/5	80.0	10/31	32.3

**Yes**	4/6	66.7	10/12	83.3	6/8	75	1/5	20.0	21/31	67.7

**HIV Knowledge levels**										

**Poor**	3/6	50.0	7/12	58.3	5/7	71.4	2/5	16.7	17/30	56.7

**Good**	3/6	50.0	5/12	41.6	2/7	28.6	3/5	60.0	13/30	43.3

*The question on religion was added to the baseline survey after piloting in the roadside village of Isangijo

81% of people reported previous HIV education from a variety of sources including schools, NGOs, churches and the community. 21/31 (71%) respondents reported previous HIV training had been provided to VAC members, with 20 of these reporting its occurrence in 2009. All participants cited the development of an HIV curriculum as important. Participants were probed about teaching methods to be incorporated into the curriculum, VAC members requested educational lectures to be included, while the use of stories in HIV education appeared unpopular.

HIV knowledge levels in the baseline survey were higher among men compared to women (Table 4), with "good" HIV knowledge levels achieved by 72.2% of males and only 33.3% of females (p = 0.041) Univariate analysis indicated previous HIV education (p = 0.002) was associated with "good" HIV knowledge levels. There was no evidence to suggest sex, village, previous uptake of VCT or previous HIV training was associated with HIV knowledge.

**Table 4:** Univariate analysis of determinants of "good" HIV knowledge amongst VAC members in the baseline survey

			Univariable analysis		
**Characteristic**	**Good knowledge**	**%**	**COR**	**95% CI**	**P value**

**Sex**					

**Female**	4/12	33.3	-	-	-

**Male**	13/18	72.2	5.2	1.07-25.31	0.041

**Village**					

**RoadsideIsangijo**	3/6	50.0	-	-	-

**Rural Welamasonga**	5/12	41.6	1.4	0.20-10.03	0.738

**Roadside Igekemaja**	2/7	28.6	2.5	0.25-24.72	0.433

**Trade-centre Kisesa**	3/5	60.0	0.67	0.06-7.35	0.741

**Previous HIV education**					

**No**	0/6	0	-	-	-

**Yes**	17/24	70.8	-	-	0.002*

**Previous HIV training**					

**No**	3/9	33.3	-	-	-

**Yes**	14/21	66.7	4	0.76-20.96	0.101

**Ever had VCT**					

**No**	3/10	30.0	-	-	-

**Yes**	14/20	70.0	5.44	1.04-28.53	0.045

*P value reported from chi square test, COR (Crude Odds Ratio) and 95% CI (confidence interval) obtained from logistic regression

All participants correctly identified that ART can prolong life, with the majority aware that ART is available free of charge within Tanzania (90%). In all villages this was much higher than the 44% of community members who knew that ART is free in Tanzania during the 2007 serosurvey (Table 5). 20 individuals (67%) believed a poor diet whilst on ART accelerated death, while 8 people (27%) believed condoms contained HIV and a further 9 (30%) unsure of the correct answer. Three respondents (10%) stated that traditional healers could cure HIV/AIDS.

**Table 5:** Comparison between village residents and VAC participants' knowledge, prior to training, on the provision of free ART within Tanzania

Village Name	Isangijo		Welamasonga		Igekemaja		Kisesa			
	**n**	**%**	**n**	**%**	**n**	**%**	**n**	**%**	**Total**	**%**

**VAC Survey: ART free**	6	-	12	-	7	-	5	-	30	-

**No**	0	0	2	16.7	1	14.3	0	0	3	10.0

**Yes**	6	100	10	83.3	6	85.7	5	100	27	90.0

**Don't Know**	0	0	0	0	0	0	0	0	0	0

**Serosurvey: ART free**	978	-	1529	-	819	-	1171	-	1560	-

**No**	172	17.6	201	13.2	217	26.5	200	11.3	1560	17.1

**Yes**	406	41.5	674	44.1	279	34.1	884	49.9	4002	43.9

**Don't Know**	400	40.9	654	42.8	323	39.4	687	40.9	3558	39.0

### VCT uptake amongst VAC members

Disparities in VCT uptake were evident among VAC members by village and sex. More men than women reported having ever used VCT (79% versus 50%), with this result approaching statistical significance (p = 0.09). VCT uptake was highest in the most rural village with 10/12 (83%) participants in the baseline survey reporting previous use of the service. Whilst the numbers surveyed in the trade centre were small, only 1/5 (20%) reported previous use of VCT.

### VAC in practice

When VAC members were asked about the desired frequency of meetings to discuss issues surrounding HIV, the majority (71%) suggested meetings every fortnight. The majority 21/23 (91%) stated that young people prior to sexual debut should be a priority for targeted HIV messages. A high proportion of VAC members (28/31; 90%) wanted traditional healers included in the education of the community regarding HIV, and while all women highlighted the importance of females in educating their peers, only 89% of men thought that women should be involved in HIV education. The majority of participants (93.4%) suggested the primary role of the VAC was to educate their communities on issues associated with HIV, with the exception of two individuals who reportedly did not know what the role and function of the VAC was. Other roles mentioned included providing counselling, advice and encouraging individuals to undergo HIV testing.

Methods for recruiting VAC members appeared similar across the four villages. Members were commonly selected by village leaders or appointed by existing VAC members and/or the village executive officer. One participant reported having been selected during a village meeting. Individuals' perceptions on why they had been appointed onto the VAC included respect within the community, the ability to educate people as well as believing they had good knowledge of HIV.

### Post-training survey

**Figure 1:**
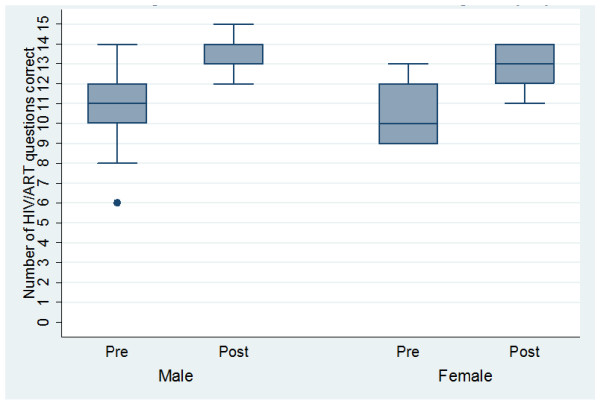
**HIV knowledge levels at the baseline and post-training surveys among VAC members by sex**.

A strong association was observed between knowledge levels in the baseline and post-training surveys, with an improvement observed across all the villages (p = < 0.001). Similarly, HIV understanding improved among both sexes, with the impact of training appearing greater in females, with significantly enhanced knowledge levels at the end of the study (p = 0.001) (Figure 1). Knowledge of HIV transmission improved between the baseline and post-training surveys, with 4/31 (13%) versus 12/29 (42%) scoring 100% on the correct routes of HIV transmission (p = 0.001). Misconceptions widely held amongst the VAC before HIV training had apparently been addressed, with all participants in the post-training survey reporting that condoms do not contain HIV (Table 6).

**Table 6:** HIV knowledge in the VACs in the baseline and post-training survey

Statement	Baseline		Post-survey	
	**n/N**	**%**	**n/N**	**%**

**ART prolongs life if taken consistently**				

True	30/30	100	29/29	100

False	0	0	0	0

Don't Know	0	0	0	0

**ART is available free of charge in Tanzania**				

True	27/30	90.0	28/29	96.6

False	3/30	10.0	0	0

Don't Know	0	0	1/29	3.4

**Inadequate nutrition whilst on ART accelerates death**				

True	20/30	66.7	27/29	93.1

False	3/30	10.0	2/29	6.9

Don't Know	7/30	23.3	0	0

**Condoms contain virus**				

True	8/30	26.7	0	0

False	13/30	43.3	29/29	100

Don't Know	9/30	30.0	0	0

**HIV is only found in the trade centre**				

True	2/30	6.7	0	0

False	28/30	93.3	29/29	100

Don't Know	0	0	0	0

**HIV symptoms can be cured by traditional/ spiritual healing**				

True	3/30	10.0	0	0

False	24/30	80.0	29/29	100

Don't Know	3/30	10.0	0	0

VACs reported that the training was useful, informative and sufficient to enable them to provide HIV education to their respective communities. All requested additional HIV training in the future, with 15 (52%) suggesting training at three-monthly intervals. 27 VAC members (93%) wanted help to facilitate village meetings for dissemination to villagers. Three topics; stigma 26/29 (90%), condom demonstrations 24/29 (83%) and sexually transmitted infections 24/29 (82.8%) were highlighted from a variety of options as topics on which the VAC would like to learn more (Table 7).

**Table 7:** Suggested topics for inclusion in a revised HIV curriculum targeting VACs

Future curriculum topic	Yes	%	No	%
**Puberty**	12	41.4	17	58.7

**Gender relations**	13	44.8	16	55.2

**Condom demonstrations**	24	82.8	5	17.2

**STIs**	24	82.8	5	17.2

**Stigma/discrimination**	26	89.7	3	10.3

**Witchcraft**	11	37.9	18	62.1

**Total**	29	100	29	100

## Discussion

Although VACs are ideally placed to disseminate HIV knowledge to their communities, this study highlighted two main obstacles hampering their efforts to do so: (i) poor knowledge demonstrated by VAC members in the baseline survey and (ii) the composition of the VACs.

Nevertheless, this pilot study clearly suggests that providing VACs with HIV education and training can increase HIV knowledge levels. The post-training surveys provided positive results of the curriculum's effectiveness, illustrating that fairly short educational sessions can result in an immediate increase in knowledge. Furthermore, the baseline surveys highlighted that VAC members who had received previous HIV education had better HIV knowledge levels, indicating that knowledge can be retained, but does need reinforcing. These findings encourage the development of a system of continuing education for the VAC in order to improve and maintain good knowledge of HIV and ART. Education needs to be appropriate to the cultural context, and may vary across the country where different beliefs, behaviours and cultural practices exist. The curriculum incorporated participatory learning action techniques to encourage learning and engage VAC members with the trainer.

A key success of this pilot curriculum was the observed change in belief surrounding condoms. Prior to HIV training, 57% of participants believed that unused condoms contain the HIV virus compared to none in the follow-up survey. Kisesa is not isolated in these beliefs surrounding condoms, with similar reports documented in other regions of Tanzania [13]. For condom interventions to be successful, negative beliefs and attitudes need to be addressed with accurate information.

Univariate analysis of the baseline survey revealed initial knowledge levels to be lower amongst female members of the VAC (p = 0.041). This concurs with results from other studies in rural Tanzania, which have illustrated that girls in school have significantly poorer HIV/AIDS knowledge than boys [26]. Boys and men tend to have greater exposure to HIV information than girls, which is likely to explain their more accurate knowledge on average, about the virus. Exposure to such information may come from within and outside the classroom, including through entertainment education or public awareness campaigns. Adolescent women in poor rural communities have few opportunities to access television or radio, and often have less access to schooling and lower levels of educational attainment [26]. It is therefore particularly important that female VAC members are well trained about HIV, and are encouraged to provide their contemporaries in the community with general health education and HIV prevention information, as well as ways to negotiate safe sexual behaviour. It may be appropriate on occasion for the VAC to provide female-only educational sessions, which may help to reduce or eliminate the HIV knowledge disparities between the sexes that are evident across the continent [26].

This study suggested that knowledge regarding the free availability of ART in Tanzania was higher amongst VAC members compared with the general community. However the discrepancy could be attributed to the fact that the data from the general community were collected several years earlier in 2007, when there was less knowledge about ART across the whole community. As free treatment only became available in Mwanza city in 2005 and within Kisesa health centre in 2008, it is likely that awareness about this treatment has increased over time.

VAC guidelines promote recruitment of a diverse array of individuals from across the community [15,16]. However, the composition of the VACs in Kisesa indicated a predominantly male committee, averaging 40 years of age with the absence of youths, elders, teachers and religious representatives. This imbalance may reduce opportunities for dissemination of HIV education, as opportunities to interact with youths and women within these communities may not be available. In addition, the absence of teachers among the committees in Kisesa may lead to missed opportunities for HIV education within schools, as primary school teachers can be trained to deliver HIV education messages [27]. The MKV programme rolled out in rural Mwanza implemented peer-assisted methods of education in addition to teacher-led education. In MKV peer educators successfully created a drama serial to aid in promoting desirable behaviours [17]. Written curricula, which can be implemented outside the school environment in a community setting can potentially reach higher risk youths who have dropped out of school [21]. Recruitment of youths onto the VAC may provide opportunity to educate and inform similar age groups about HIV; especially those high-risk individuals.

Tanzanian guidelines also advise that people living with HIV/AIDS (PLHA) are recruited onto VACs [15], although it was not possible to determine from this study whether this has been adopted within the VACs of Kisesa. As religion is highly influential in Tanzanian society, incorporating faith leaders into VACs is desirable, particularly since religious leaders often influence people's beliefs and behaviours as well as offering support and care for PLHA [7,28]. In particular, previous studies have shown that some PLHA gain support from their religion, helping them to live positively with HIV and providing them with courage to face their condition [7]. Providing religious leaders with education, through membership on the VAC could allow them to encourage their followers to access services for testing and care, although it is important that religious leaders emphasise that prayer and medical care can be combined for PLHA [7].

The turn-over of VAC recruits was most apparent in the trade-centre and roadside villages. Accordingly, to accommodate this faster turnover of recruits, HIV education may need to be delivered more frequently to VAC members in villages that are closer to urban areas, compared to the more remote villages, in order to ensure that each new member is sufficiently informed.

Several steps were taken to address the potential for bias in the results. In order to limit selection bias, invitations were distributed to every member of the VAC several days prior to training. However participation in the trade centre was lowest, which may be explained by members of this VAC being more likely to have other work commitments compared to those in the more rural areas. Social desirability bias may have resulted in an over-estimate of the success of the intervention, if participants reported what they felt the interviewer would like to hear, particularly in the post-training survey. To reduce this bias as far as possible, a research assistant who had previously established a rapport with these individuals, conducted both the baseline and post-training surveys after briefing the participants on the purpose of the study and explaining that their responses would be anonymous.

The HIV curriculum was translated from English to Kiswahili by a native Kiswahili research assistant fluent in English, however some words could not be directly translated. To aid in this process as much as possible, both the KiSwahili and English versions of MKV were used as a reference tool.

This pilot study is limited by the small sample size. Although every effort was made to encourage full participation at each stage of the study, the maximum attendance at each stage was 45 members from 3 VACs. Expanding the evaluation with more VACs across Tanzania would increase the sample size and should be considered in the future. There is also further scope to expand this project in the future. Whilst the pilot study evaluated the success of the HIV training within the VAC, no evaluation was conducted to establish if this knowledge had been disseminated to the community at large. In addition, monitoring and evaluation is ideally required at the community level to observe if improved HIV knowledge is translated into behavioural change and increased uptake of HIV services available to residents of Kisesa ward.

## Conclusion

In conclusion, VACs are ideally placed in the community to provide HIV education. It is essential that comprehensive HIV education and training is scaled up across Tanzania to provide VAC members with the knowledge to disseminate to the community. It is important to ensure that recruitment is in line with Tanzanian policy, to maximise opportunities for VAC members to interact with peers and groups within the community. In Kisesa, the VAC needs to diversify to include youths, religious leaders, teachers and PLHA and create an equal gender balance. Further work should assess VAC existence and composition in other regions of Tanzania.

Providing simple clear messages which address misconceptions demonstrated that HIV knowledge levels could be enhanced in the short-term. Expansion of the curriculum, going beyond HIV transmission, prevention and ART, to include issues of stigma and gender relations, needs to be explored. Further studies to evaluate how long this knowledge within the VAC is retained are required, in order to calculate timing intervals for future training sessions. As the role of the VAC is to provide HIV education, evaluative studies need to be conducted to assess if the VAC's knowledge is being disseminated to the wider community, and resulting in improved uptake of HIV prevention and treatment services within Kisesa ward.

## Competing interests

The authors declare that they have no competing interests.

## Authors' contributions

EE, AW, JT and MU conceived the design of the study. BN conducted the fieldwork. EE wrote the first draft of the manuscript. MU, director of the research project provided overall advice and facilitated the fieldwork. All co-authors read and commented on the draft versions of the paper and participated in the editing process. All co-authors have read and approved the final manuscript.

## Pre-publication history

The pre-publication history for this paper can be accessed here:

http://www.biomedcentral.com/1472-698X/11/14/prepub

## References

[B1] Wringe A Roura M Urassa M Busza J Athanas V Zaba B Doubts, denial and divine intervention: understanding delayed attendance and poor retention rates at a HIV treatment programme in rural Tanzania. [Internet] AIDS care 2009 21 5 632 7 [cited 2011 May 30] Available from: http://www.ncbi.nlm.nih.gov/pubmed/19444672 10.1080/09540120802385629 19444672

[B2] Falnes EF Tylleskär T de Paoli MM Manongi R Engebretsen IMS Mothers' knowledge and utilization of prevention of mother to child transmission services in northern Tanzania. [Internet] Journal of the International AIDS Society 2010 1336 [cited 2011 May 30] Available from: http://www.ncbi.nlm.nih.gov/pubmed/20840784 10.1186/1758-2652-13-36 PMC3161341 20840784

[B3] Roura M Urassa M Busza J Mbata D Wringe A Zaba B Scaling up stigma? The effects of antiretroviral roll-out on stigma and HIV testing. Early evidence from rural Tanzania. [Internet] Sexually transmitted infections 2009 85 4 308 12 [cited 2010 Nov 29] Available from: http://www.pubmedcentral.nih.gov/articlerender.fcgi?artid=2708343&tool=pmcentrez&rendertype=abstract 10.1136/sti.2008.033183 19036776 PMC2708343

[B4] Remes P Renju J Nyalali K Medard L Kimaryo M Changalucha J Obasi A Wight D Dusty discos and dangerous desires: community perceptions of adolescent sexual and reproductive health risks and vulnerability and the potential role of parents in rural Mwanza, Tanzania. [Internet] Culture, health & sexuality 2010 12 3 279 92 [cited 2011 May 30] Available from: http://www.ncbi.nlm.nih.gov/pubmed/19941178 10.1080/13691050903395145 19941178

[B5] Parker R Aggleton P HIV and AIDS-related stigma and discrimination: a conceptual framework and implications for action. [Internet] Social science & medicine (1982) 2003 57 1 13 24 [cited 2011 May 30] Available from: http://www.ncbi.nlm.nih.gov/pubmed/12753813 10.1016/S0277-9536(02)00304-0 12753813

[B6] Wolfe WR Weiser SD Bangsberg DR Thior I Makhema JM Dickinson DB Mompati KF Marlink RG Effects of HIV-related stigma among an early sample of patients receiving antiretroviral therapy in Botswana. [Internet] AIDS care 2006 18 8 931 3 [cited 2011 May 30] Available from: http://www.ncbi.nlm.nih.gov/pubmed/17012082 10.1080/09540120500333558 17012082

[B7] Roura M Nsigaye R Nhandi B Wamoyi J Busza J Urassa M Todd J Zaba B "Driving the devil away": qualitative insights into miraculous cures for AIDS in a rural Tanzanian ward. [Internet] BMC public health 2010 10427 [cited 2011 May 30] Available from: http://www.ncbi.nlm.nih.gov/sites/entrez/20646300?dopt=Abstract&holding=f1000,f1000m,isrctn 10.1186/1471-2458-10-427 PMC2916904 20646300

[B8] Plummer M Mshana G Wamoyi J Shigongo Z Hayes R Ross D Wight D "The man who believed he had AIDS was cured": AIDS and sexually-transmitted infection treatment-seeking behaviour in rural Mwanza, Tanzania [Internet] AIDS Care 2006 18 5 460 466 [cited 2011 Feb 5] Available from: http://www.ncbi.nlm.nih.gov/pubmed/16777638 10.1080/09540120500220367 16777638

[B9] Maman S Abler L Parker L Lane T Chirowodza A Ntogwisangu J Srirak N Modiba P Murima O Fritz K A comparison of HIV stigma and discrimination in five international sites: the influence of care and treatment resources in high prevalence settings. [Internet] Social science & medicine (1982) 2009 68 12 2271 8 [cited 2011 Apr 19] Available from: http://www.pubmedcentral.nih.gov/articlerender.fcgi?artid=2696587&tool=pmcentrez&rendertype=abstract 10.1016/j.socscimed.2009.04.002 19394121 PMC2696587

[B10] Roura M Busza J Wringe A Mbata D Urassa M Zaba B Barriers to sustaining antiretroviral treatment in Kisesa, Tanzania: a follow-up study to understand attrition from the antiretroviral program. [Internet] AIDS patient care and STDs 2009 23 3 203 10 [cited 2010 Nov 29] Available from: http://www.pubmedcentral.nih.gov/articlerender.fcgi?artid=2776987&tool=pmcentrez&rendertype=abstract 10.1089/apc.2008.0129 19866538 PMC2776987

[B11] Roura M Wringe A Busza J Nhandi B Mbata D Zaba B Urassa M "Just like fever": a qualitative study on the impact of antiretroviral provision on the normalisation of HIV in rural Tanzania and its implications for prevention. [Internet] BMC international health and human rights 2009 922 [cited 2010 Jul 22] Available from: http://www.pubmedcentral.nih.gov/articlerender.fcgi?artid=2759900&tool=pmcentrez&rendertype=abstract 10.1186/1472-698X-9-22 PMC2759900 19740437

[B12] Nsigaye R Wringe A Roura M Kalluvya S Urassa M Busza J Zaba B From HIV diagnosis to treatment: evaluation of a referral system to promote and monitor access to antiretroviral therapy in rural Tanzania. [Internet] Journal of the International AIDS Society 2009 12 1 31 [cited 2011 May 25] Available from: http://www.pubmedcentral.nih.gov/articlerender.fcgi?artid=2788344&tool=pmcentrez&rendertype=abstract 10.1186/1758-2652-12-31 19906291 PMC2788344

[B13] Plummer ML Wight D Wamoyi J Mshana G Hayes RJ Ross DA Farming with your hoe in a sack: condom attitudes, access, and use in rural Tanzania. [Internet] Studies in family planning 2006 37 1 29 40 [cited 2011 May 30] Available from: http://www.ncbi.nlm.nih.gov/pubmed/16570728 10.1111/j.1728-4465.2006.00081.x 16570728

[B14] Beckles1 Direct evidence of recent declines in HIV prevalence and incidence in a rural population open cohort in Northern Tanzania, 1994-2007 XVIII International AIDS Conference 2010 Vienna. Vienna: 2010

[B15] Guidelines for forming AIDS committees at local government level [Internet]. Dodoma 2003 Available from: http://www.moh.go.tz/documents/guidelines_aids_committees.pdf

[B16] UNGASS reporting for 2010 (Tanzania mainland and Zanzibar) [Internet]. Tanzania 2010 Available from: http://www.unaids.org/en/dataanalysis/monitoringcountryprogress/2010progressreportssubmittedbycountries/tanzania_2010_country_progress_report_en.pdf

[B17] Plummer ML Wight D Obasi AIN Wamoyi J Mshana G Todd J Mazige BC Makokha M Hayes RJ Ross DA A process evaluation of a school-based adolescent sexual health intervention in rural Tanzania: the MEMA kwa Vijana programme. [Internet] Health education research 2007 22 4 500 12 [cited 2010 Jun 28] Available from: http://www.ncbi.nlm.nih.gov/pubmed/17018767 17018767 10.1093/her/cyl103

[B18] Kaponda CPN Dancy BL Norr KF Kachingwe SI Mbeba MM Jere DL Research brief: community consultation to develop an acceptable and effective adolescent HIV prevention intervention. [Internet] The Journal of the Association of Nurses in AIDS Care: JANAC 18 2 72 7 [cited 2011 May 30] Available from: http://www.ncbi.nlm.nih.gov/pubmed/17403498 10.1016/j.jana.2007.01.001 17403498

[B19] Campbell C MacPhail C Peer education, gender and the development of critical consciousness: participatory HIV prevention by South African youth. [Internet] Social science & medicine (1982) 2002 55 2 331 45 [cited 2011 May 30] Available from: http://www.ncbi.nlm.nih.gov/pubmed/12144146 10.1016/S0277-9536(01)00289-1 12144146

[B20] Gallant M Maticka-Tyndale E School-based HIV prevention programmes for African youth. [Internet] Social science & medicine (1982) 2004 58 7 1337 51 [cited 2011 Jan 31] Available from: http://www.ncbi.nlm.nih.gov/pubmed/14759680 10.1016/S0277-9536(03)00331-9 14759680

[B21] Kirby DB Laris BA Rolleri LA Sex and HIV education programs: their impact on sexual behaviors of young people throughout the world. [Internet] The Journal of adolescent health: official publication of the Society for Adolescent Medicine 2007 40 3 206 17 [cited 2011 May 30] Available from: http://www.ncbi.nlm.nih.gov/pubmed/17321420 10.1016/j.jadohealth.2006.11.143 17321420

[B22] Ahmed N Flisher AJ Mathews C Jansen S Mukoma W Schaalma H Process evaluation of the teacher training for an AIDS prevention programme. [Internet] Health education research 2006 21 5 621 32 [cited 2011 May 30] Available from: http://www.ncbi.nlm.nih.gov/pubmed/16740671 10.1093/her/cyl031 16740671

[B23] Kohi TW Portillo CJ Safe J Okonsky J Nilsson AC Holzemer WL The Tanzania HIV/AIDS nursing education (THANE) preservice curriculum. [Internet] The Journal of the Association of Nurses in AIDS Care: JANAC 21 2 92 8 [cited 2011 May 30] Available from: http://www.ncbi.nlm.nih.gov/pubmed/19822445 10.1016/j.jana.2009.06.006 19822445

[B24] Renju J Nyalali K Andrew B Kishamawe C Kimaryo M Remes P Changalucha J Obasi A Scaling up a school-based sexual and reproductive health intervention in rural Tanzania: a process evaluation describing the implementation realities for the teachers. [Internet] Health education research 2010 25 6 903 16 [cited 2011 May 30] Available from: http://www.ncbi.nlm.nih.gov/pubmed/20670997 10.1093/her/cyq041 20670997

[B25] Wambura M Urassa M Isingo R Ndege M Marston M Slaymaker E Mngara J Changalucha J Boerma TJ Zaba B HIV Prevalence and Incidence in Rural Tanzania [Internet] JAIDS Journal of Acquired Immune Deficiency Syndromes 2007 46 5 616 623 [cited 2011 May 30] Available from: http://www.pubmedcentral.nih.gov/articlerender.fcgi?artid=2842883&tool=pmcentrez&rendertype=abstract 10.1097/QAI.0b013e31815a571a PMC2842883 18043316

[B26] Vavrus F Girls' schooling in Tanzania: the key to HIV/AIDS prevention? [Internet] AIDS care 2006 18 8 863 71 [cited 2010 Aug 13] Available from: http://www.ncbi.nlm.nih.gov/pubmed/17012074 10.1080/09540120500307776 17012074

[B27] Plummer ML Wight D Wamoyi J Nyalali K Ingall T Mshana G Shigongo ZS Obasi A Ross DA Are schools a good setting for adolescent sexual health promotion in rural Africa? A qualitative assessment from Tanzania. [Internet] Health education research 2007 22 4 483 99 [cited 2011 Feb 5] Available from: http://www.ncbi.nlm.nih.gov/pubmed/17018766 17018766 10.1093/her/cyl099

[B28] Watt MH Maman S Jacobson M Laiser J John M Missed opportunities for religious organizations to support people living with HIV/AIDS: findings from Tanzania. [Internet] AIDS patient care and STDs 2009 23 5 389 94 [cited 2011 May 30] Available from: http://www.ncbi.nlm.nih.gov/pubmed/19335171 10.1089/apc.2008.0195 19335171 PMC3521158

